# Comparison of acute and chronic myocardial injury in noncardiac surgical patients

**DOI:** 10.1371/journal.pone.0234776

**Published:** 2020-07-02

**Authors:** Jungchan Park, Kwangmo Yang, Seung-Hwa Lee, Jong Hwan Lee, Jeong Jin Min, Ji-hye Kwon, Ah Ran Oh, Junghyun Yeo, Jihoon Kim, Jin-ho Choi, Sang-Chol Lee, Hyeon-Cheol Gwon, Kyunga Kim, Joonghyun Ahn, Sangmin Maria Lee

**Affiliations:** 1 Department of Anesthesiology and Pain Medicine, Samsung Medical Center, Sungkyunkwan University School of Medicine, Seoul, Korea; 2 Center for Health Promotion, Samsung Medical Center, Sungkyunkwan University School of Medicine, Seoul, Korea; 3 Division of Cardiology, Department of Medicine, Heart Vascular Stroke Institute, Samsung Medical Center, Sungkyunkwan University School of Medicine, Seoul, Korea; 4 Department of Emergency Medicine, Samsung Medical Center, Sungkyunkwan University School of Medicine, Seoul, Republic of Korea; 5 Statistics and Data Center, Research Institute for Future Medicine, Samsung Medical Center, Seoul, Korea; 6 Department of Digital Health, SAIHST, Sungkyunkwan University, Seoul, Korea; Cleveland Clinic, UNITED STATES

## Abstract

**Purpose:**

Perioperative myocardial injury is a predictor of postoperative mortality, but the clinical impact of chronic injury during the perioperative period has not been fully investigated. This study aimed to evaluate chronic myocardial injury during the perioperative period in comparison with normal and acute myocardial injury.

**Methods:**

Patients with serial cardiac troponin measurements before and within 30 days following noncardiac surgery were divided into three groups: normal, acute injury, and chronic injury groups. Acute and chronic myocardial injuries were stratified according to 2018 recommendations by the International Federation of Clinical Chemistry and Laboratory Medicine’s Task Force on Clinical Applications of Bio-Markers. Thirty-day and one-year mortalities after surgery were compared.

**Results:**

Of the 22,969 patients reviewed, 17,671 (76.9%) were classified into the normal, 5,179 (22.5%) into the acute injury, and 119 (0.5%) into the chronic injury groups. The acute and chronic injury groups had higher 30-day mortalities compared with the normal group (0.8% vs. 8.0%; hazard ratio [HR], 11.00; 95% confidence interval [CI], 9.05–13.37; P < 0.001 and 0.8% vs. 7.6%; HR, 10.55; 95% CI, 5.37–20.72; P < 0.001, respectively). In a direct comparison between the acute and chronic injury groups using an inverse probability of weighting adjustments, the 30-day and one-year mortalities were not significantly different.

**Conclusion:**

Chronic myocardial injury during the perioperative period may show similar clinical impacts on postoperative mortality compared with acute injury. Further studies are needed.

## Introduction

Myocardial injury is defined as any elevation of cardiac troponin (cTn) above the 99^th^ percentile upper reference limit (URL) [1], and has shown significant associations with adverse outcomes [2]. In surgical patients, this association was consistent [3], but the differential diagnoses for elevated cTn can be broad. As ischemic myocardial injury after noncardiac surgery was identified as a strong independent predictor of mortality, the diagnostic criteria for perioperative myocardial injury excluded chronic injury [4–6], and the following guidelines recommend serial measurement of cTn during the perioperative period [7, 8]. A recent study also demonstrated that an increase in absolute cTn value over the 99^th^ percentile URL during the perioperative period was associated with mortality [9], but the differentiation between acute and chronic conditions still remains challenging [10]. Although an elevation of cTn beyond the 99th percentile is generally considered an acute state when the initial level was below the URL, no standard exists for what degree of cTn rise or fall should be identified as acute myocardial injury when the initial level was already above the URL [10]. Therefore, chronic myocardial injury conditions in surgical patients have been hard to investigate.

Based on the International Federation of Clinical Chemistry and Laboratory Medicine’s Task Force on Clinical Applications of Bio-Markers, criteria for identifying acute myocardial injury was recently proposed as when the initial cTn value is greater than the 99th percentile of the URL: an increase of at least 50% of the 99th percentile value or a change greater than 20% relative to the initial value [10, 11]. In this study, we stratified myocardial injury into acute and chronic conditions during the perioperative period. The mortalities of these patients were compared according to theses conditions and to patients without perioperative myocardial injury. Our study may provide valuable information on clinical outcomes of patients who had chronic myocardial injury and were not adequately diagnosed with perioperative myocardial injury after undergoing noncardiac surgery.

## Methods

This was a single-center observational study using data from the SMC-TINCO registry (Samsung Medical Center Troponin in Noncardiac Operation), which is a large, de-identified cohort. The approval for this study and the requirement for written informed consent for access to the registry were waived by the Institutional Review Board at Samsung Medical Center because the entire dataset was initially extracted in de-identified form (SMC 2019-08-048). The SMC-TINCO was registered at https://cris.nih.go.kr before patient enrollment (Clinical Trial Registration: KCT0004244).

### Study population

The SMC-TINCO registry contains 43,019 consecutive patients who, from January 2010 to June 2019, had at least one measured cTn I during preoperative evaluation or within 30 days after noncardiac surgery at Samsung Medical Center, Seoul, Korea. Our institution uses a paperless medical record system that contains data from over 4 million patients and more than 2 million surgeries, 900 million laboratory findings, and 200 million prescriptions. All data in this registry were extracted using the “Clinical Data Warehouse Darwin-C” of Samsung Medical Center, an electronic system built for investigators to search and retrieve de-identified medical records from the institutional electronic archive system. Mortality statistics from institutions other than our own were consistently validated with and updated from the National Population Registry of the Korea National Statistical Office using a unique personal identification number when available. From this registry, we enrolled adult patients with serial cTn measurements within 30 days before and after surgery. We excluded patients who received cardiac compression before the follow-up cTn measurement to rule out an elevation from mechanical injury. Enrolled patients were divided into normal, acute injury, and chronic injury groups according to recent guidelines [11]. Considering the large difference in the number of patients in each group and the limited potential of statistical adjustment to permit comparison across the three groups, acute and chronic injuries were directly compared using an additional statistical adjustment.

### Definitions & outcome measures

Chronic myocardial injury was defined as initial cTn level above the 99th percentile of the URL and an absolute increase in any follow-up values that did not exceed 50% of the 99th percentile value or if relative changes were smaller than 20% of the initial value [10, 11]. The primary outcome was 30-day mortality, and one-year mortality was also compared. Mortality was classified into cardiovascular and noncardiovascular mortalities, and cardiovascular mortality was defined as death related to myocardial infarction, cardiac arrhythmia, heart failure, stroke, or vascular causes. Noncardiovascular mortality was defined as death from a cause other than cardiovascular conditions. All deaths without an undisputed noncardiovascular cause were considered cardiovascular deaths [12]. Active cancer was defined as histologic cancer diagnosis within the past six months [13]. High-risk surgery was defined according to the 2014 European Society of Cardiology/Anesthesiology guidelines [14].

### Perioperative cTn I measurement & management

Perioperative management followed institutional protocols that were based on current guidelines. According to the protocol, preoperative cTn measurement was recommended for patients with moderate to high cardiovascular risk such as history of ischemic heart disease, heart failure, stroke including transient ischemic attack, diabetes mellitus on insulin therapy, or chronic kidney disease or for patients undergoing moderate- to high-risk surgeries as identified by perioperative guidelines [14]. For postoperative follow-up evaluation was conducted considering individual risks such as suspected symptoms of ischemic disease. For patients with mild risk, cTn was measured at the attending clinician’s discretion. An automated analyzer (Advia Centaur XP; Siemens Healthcare Diagnostics, Erlangen, Germany) using a highly sensitive immunoassay was used. The lowest limit of detection was 6 ng/L, and the 99^th^ percentile upper reference limit (URL) was 40 ng/L, as provided by the manufacturer. Patients with elevated cTn levels were referred to cardiologists for further evaluation and proper management. Other perioperative management followed our institutional protocols, which are based on current guidelines.

### Statistical analyses

Analysis of variance (ANOVA) or Kruskal-Wallis tests were used to compare differences in baseline characteristics of the three groups, as applicable. In two-group comparisons, differences were compared by the t-test or the Mann-Whitney test, and presented as means ± standard deviations (SD) or medians with interquartile ranges (IQR) for continuous variables. Categorical variables were presented as numbers with percentages and compared using the chi-square or Fisher’s exact tests. Kaplan-Meier estimates were used to generate survival curves and results were compared with the log-rank test. Cox regression was used to compare outcomes and the Bonferroni correction was applied in the three-group comparisons. In the two-group comparisons, we used weighted regression models with inverse probability weighting (IPW) to further reduce selection bias while maintaining balanced confounding variables [15]. According to this technique, weights for patients with chronic injury were the inverse of the propensity score, and weights for patients with acute injury were the inverse of the propensity score. Outcomes were compared using a stratified Cox regression model and were reported as adjusted hazard ratios (HR) with 95% confidence intervals (CI). We estimated the potential impact of unmeasured confounders to evaluate the sensitivity of our results [16]. Spearman's rank correlation was used to calculate the study power regarding the sample size [17]. The power of analysis was 0.99 when HR was 2.0 in the comparison between the normal and the acute group, and in the comparison between the normal and the group the power was 0.41 and when HR was 0.6. Statistical analyses were performed with R 3.6.3 (Vienna, Austria; http://www.R-project.org/). All tests were two-tailed, and *P* < 0.05 was considered statistically significant.

## Results

### Entire population

**Fig 1:**
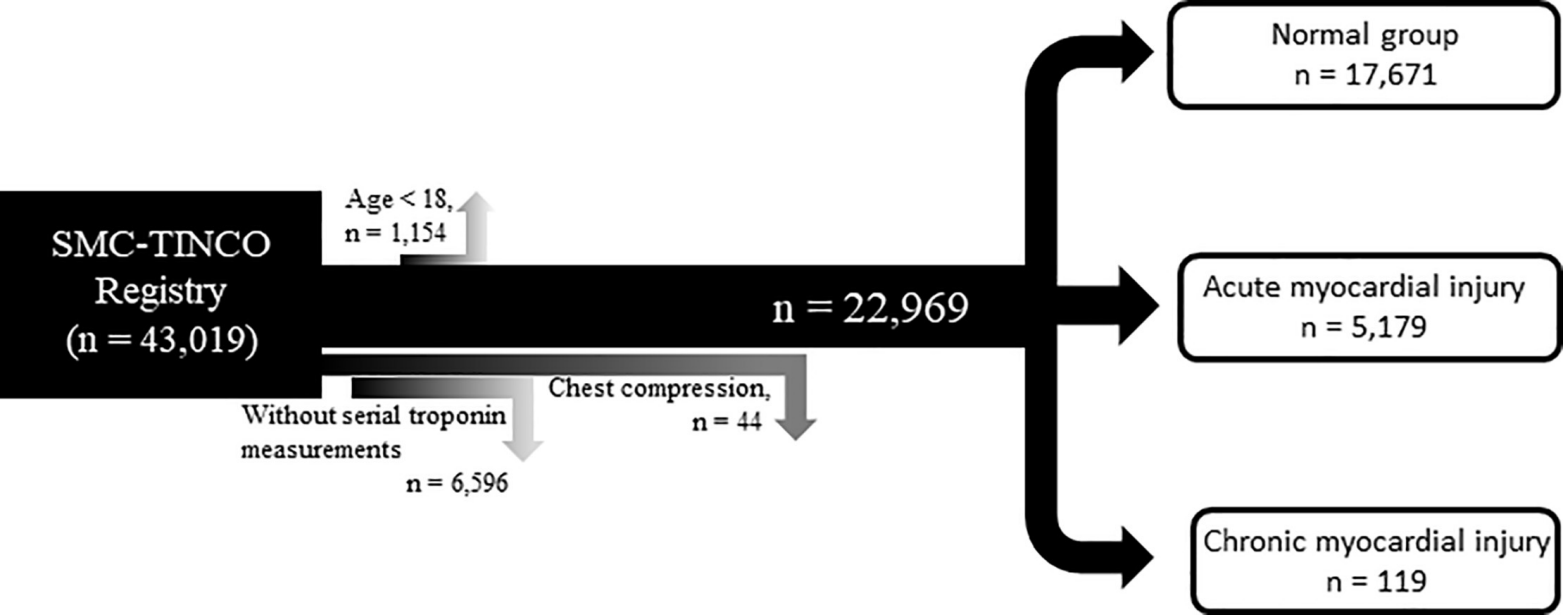
Patient flowchart.

We identified 23,013 patients with multiple cTn measurements before and within 30 days after surgery. After excluding 44 patients who received cardiac massage before cTn measurements, 22,969 patients were left for analysis. The patients were divided into three groups: 17,671 (76.9%) normal patients, 5,179 (22.5%) acute injury patients, and 119 (0.5%) chronic injury patients (Fig 1). The baseline characteristics of the entire population are presented in Table 1. Patients with chronic injuries tended to have undergone lower risk surgeries with shorter operative times compared with normal and acute injury patients. The types of surgeries are summarized in S1 Table, available as Electronic Supplementary Material.

**Table 1:** Baseline characteristics of the entire population.

	Normal	Acute myocardial injury	Chronic myocardial injury	p-value
	(N = 17671)	(N = 5179)	(N = 119)	
Male	10269 (58.1)	3175 (61.3)	74 (62.2)	<0.001
Age	63.4 (±12.9)	65.7 (±13.8)	64.1 (±14.0)	<0.001
Diabetes	9784 (55.4)	2867 (55.4)	70 (58.8)	0.75
Hypertension	10413 (58.9)	3484 (67.3)	83 (69.7)	<0.001
Current smoking	1837 (10.4)	483 (9.3)	4 (3.4)	<0.001
Current alcohol	3578 (20.2)	764 (14.8)	15 (12.6)	0.004
Chronic kidney disease	754 (4.3)	719 (13.9)	28 (23.5)	<0.001
History of ischemic heart disease	2931 (16.6)	1277 (24.7)	25 (21.0)	<0.001
History of heart failure	379 (2.1)	201 (3.9)	8 (6.7)	<0.001
History of stroke	1265 (7.2)	508 (9.8)	17 (14.3)	<0.001
History of arrhythmia	1296 (7.3)	549 (10.6)	12 (10.1)	<0.001
History of heart valve disease	245 (1.4)	107 (2.1)	3 (2.5)	0.002
Active cancer	8646 (48.9)	2033 (39.3)	45 (37.8)	<0.001
Preoperative care				
Intensive care unit	541 (3.1)	610 (11.8)	9 (7.6)	<0.001
ECMO	0	1 (0.0)	0	0.18
Continuous renal replacement therapy	16 (0.1)	61 (1.2)	0	<0.001
Ventilator	70 (0.4)	140 (2.7)	2 (1.7)	<0.001
Operative variables				
ESC/ESA surgical high risk	4370 (24.7)	1381 (26.7)	11 (9.2)	<0.001
Emergency operation	2247 (12.7)	1490 (28.8)	36 (30.3)	<0.001
General anesthesia	16130 (91.3)	4514 (87.2)	95 (79.8)	<0.001
Operation duration, hours	3.30 (±2.12)	3.53 (±2.79)	2.52 (±2.11)	
Continuous infusion of inotropics	4562 (25.8)	2217 (42.8)	37 (31.1)	<0.001
RBC transfusion	1321 (7.5)	826 (15.9)	9 (7.6)	<0.001

Data are presented as n (%) or mean (±standard deviation).

ECMO = extracorporeal membranous oxygenation; RAAS = renin-angiotensin-aldosterone system; ESC = European Society of cardiology; ESA = European Society of Anaesthesiology; RBC, red blood cell.

**Fig 2:**
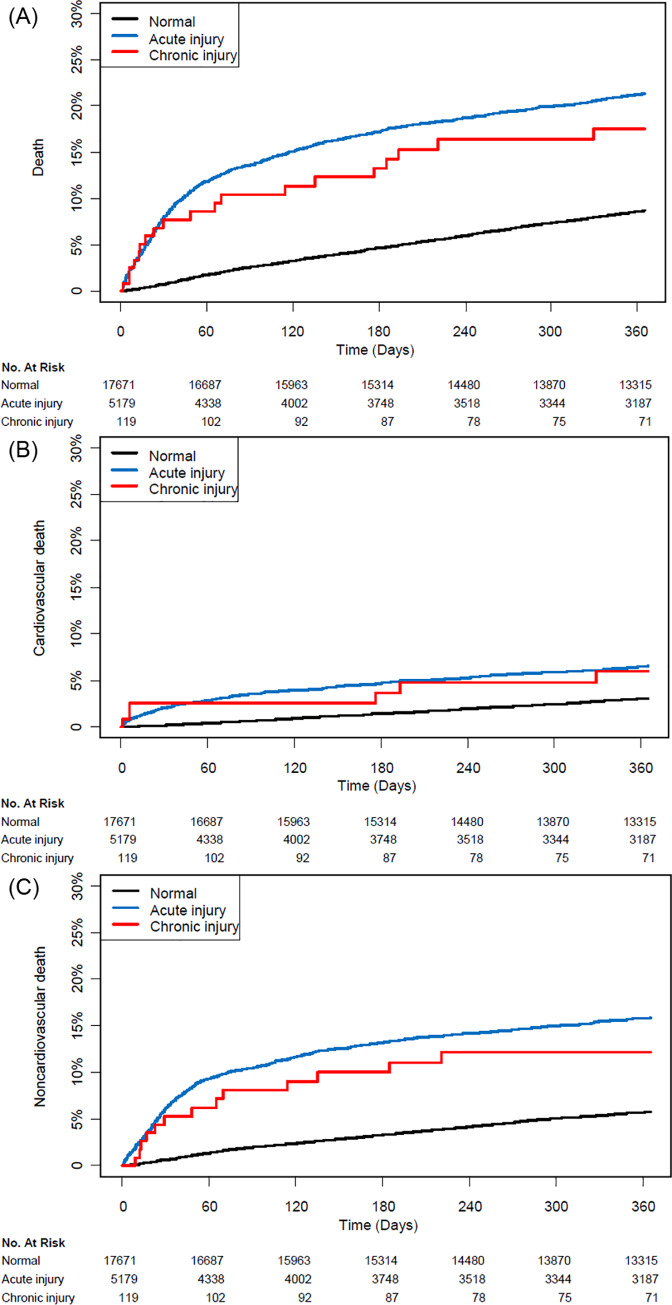
Kaplan-Meier curves of mortalities for the three groups.

As we used mortality data validated from the National Population Registry of the Korea National Statistical Office, all study patients were followed up for one year without drop out. Compared with the normal group, the acute and chronic groups both showed significantly higher 30-day mortalities (0.8% vs. 8.0%; HR, 11.00; 95% CI, 9.05–13.37; *P* < 0.001 and 0.8% vs. 7.6%; HR, 10.55; 95% CI, 5.37–20.72; *P <* 0.001, respectively), and the results were consistent regardless of cause of death. One-year mortalities were also higher in the acute injury group (7.9% vs. 20.0%; HR, 2.41; 95% CI, 2.21–2.63; *P* < 0.001) and in the chronic injury group (7.9% vs. 16.0%; HR, 2.21; 95% CI, 1.40–3.49; *P* < 0.001) compared with the normal group, but cardiovascular mortality during one-year follow-up was not significantly higher for the chronic injury group (2.7% vs. 5.0%; HR, 2.05; 95% CI, 0.91–4.63; *P* = 0.25; Table 2). The survival curves are shown in Fig 2.

**Table 2:** Mortalities of the entire population.

	Normal	Acute myocardial injury	Chronic myocardial injury
	(N = 17671)	(N = 5179)	(N = 119)
30-day mortality, No (%)	134 (0.8)	415 (8.0)	9 (7.6)
Unadjusted HR (95% CI)	1 [reference]	11.0 (9.05–13.37)	10.55 (5.37–20.72)
p-value		<0.001	<0.001
*Adjusted HR (95% CI)		7.73 (6.29–9.51)	5.74 (2.84–11.61)
p-value		<0.001	<0.001
Cardiovascular mortality, No (%)	28 (0.2)	102 (2.0)	3 (2.5)
Unadjusted HR (95% CI)	1 [reference]	12.85 (8.46–19.52)	16.66 (5.07–54.81)
p-value		<0.001	<0.001
*Adjusted HR (95% CI)		8.20 (5.27–12.76)	9.78 (2.90–36.03)
p-value		<0.001	0.001
Non-cardiovascular mortality, No (%)	106 (0.6)	313 (6.0)	6 (5.0)
Unadjusted HR (95% CI)	1 [reference]	10.5 (8.43–13.09)	8.91 (3.91–20.28)
p-value		<0.001	<0.001
*Adjusted HR (95% CI)		7.57 (5.99–9.56)	4.92 (2.09–11.55)
p-value		<0.001	0.001
One-year mortality, No (%)	1403 (7.9)	1037 (20.0)	19 (16.0)
Unadjusted HR (95% CI)	1 [reference]	2.88 (2.65–3.12)	2.35 (1.50–3.71)
p-value		<0.001	<0.001
*Adjusted HR (95% CI)		2.41 (2.21–2.63)	2.21 (1.40–3.49)
p-value		<0.001	0.002
Cardiovascular mortality, No (%)	474 (2.7)	285 (5.5)	6 (5.0)
Unadjusted HR (95% CI)	1 [reference]	2.37 (2.04–2.74)	2.23 (1.00–4.98)
p-value		<0.001	0.15
*Adjusted HR (95% CI)		1.93 (1.65–2.26)	2.05 (0.91–4.63)
p-value		<0.001	0.25
Non-cardiovascular mortality, No (%)	929 (5.3)	752 (14.5)	13 (10.9)
Unadjusted HR (95% CI)	1 [reference]	3.13 (2.85–3.45)	2.42 (1.40–4.19)
p-value		<0.001	0.005
*Adjusted HR (95% CI)		2.65 (2.39–2.94)	2.31 (1.33–4.01)
p-value		<0.001	0.009

### Acute vs. chronic myocardial injury

Among 5,298 patients with perioperative myocardial injury, 5,179 (97.8%) injuries were acute and 119 (2.2%) were chronic. In a direct comparison between the two groups, the incidence of high-risk surgery, the need for red blood cell transfusion, and operative time was higher in the acute injury group, while the chronic group showed a higher incidence of chronic kidney disease, general anesthesia, and need for inotropic drug infusion (S2 Table, available as Electronic Supplementary Material). The mortalities between the two groups did not differ, regardless of follow-up period or cause of death (8.0% vs. 7.6%; HR, 1.33; 95% CI, 0.75–2.37; *P* = 0.33 for 30-day mortality and 20.0% vs. 16.0%; HR, 0.85; 95% CI, 0.54–1.32; *P* = 0.47; Table 3).

**Table 3:** Mortalities of the patients with perioperative myocardial injury.

			Univariate analysis		Multivariate analysis		IPW analysis	
	Acute myocardial injury	Chronic myocardial injury	Unadjusted HR (95% CI)	p-value	Adjusted HR (95% CI)	p-value	Adjusted HR (95% CI)	p-value
	(N = 5179)	(N = 119)						
30-day mortality	415 (8.0)	9 (7.6)	0.95 (0.49–1.85)	0.89	0.96 (0.49–1.86)	0.9	1.33 (0.75–2.37)	0.33
Cardiovascular death	102 (2.0)	3 (2.5)	1.29 (0.41–4.07)	0.66	1.43 (0.45–4.57)	0.54	0.79 (0.17–3.60)	0.76
Noncardiovascular death	313 (6.0)	6 (5.0)	0.84 (0.38–1.89	0.68	0.83 (0.37–1.87)	0.65	1.50 (0.80–2.79)	0.2
One-year mortality	1037 (20.0)	19 (16.0)	0.80 (0.51–1.26)	0.34	0.83 (0.53–1.31)	0.43	0.85 (0.54–1.32)	0.47
Cardiovascular death	285 (5.5)	6 (5.0)	0.92 (0.41–2.07)	0.84	0.98 (0.43–2.21)	0.96	0.52 (0.17–1.56)	0.24
Noncardiovascular death	752 (14.5)	13 (10.9)	0.76 (0.44–1.31)	0.32	0.79 (0.45–1.36)	0.39	0.96 (0.59–1.57)	0.88

IPW = inverse probability weighting.

### Sensitivity analysis

Sensitivity of the effect of an unmeasured confounder with an assumed prevalence of 40% on the observed association was calculated, and the association between 30-day mortality and chronic myocardial injury compared with the normal group was consistently significant under any circumstances (S3 Table, available as Electronic Supplementary Material).

## Discussion

The results of this study showed that, in surgical patients with myocardial injury, acute injury incidence was overwhelmingly higher than chronic injury incidence. The risk of mortality was higher in both the acute and chronic injury groups compared with the normal group, and the risk level between the two injury categories was not significantly different. Our study suggests that chronic myocardial injury as well as acute injury is associated with mortality after noncardiac surgery.

Perioperative myocardial injury after noncardiac surgery has become the strongest predictor and one of the most common postoperative states associated with mortality [4–6, 9]. Despite recent studies focused on prevention and treatment of acute myocardial injury [4, 18, 19], criteria for acute and chronic conditions after perioperative myocardial injury have not been fully established [10]. Generally, a rise or fall that exceeds the biological or analytical variation in cTn is regarded as an acute condition [20]. Previously, in patients with acute myocardial infarction, the larger rise or fall in cTn concentration yielded a higher positive predictive value, and absolute rather than relative changes have shown higher diagnostic accuracy [21, 22]. For this study, we applied a recently proposed criteria using both absolute and relative cTn changes based on the latest recommendation of the International Federation of Clinical Chemistry and Laboratory Medicine’s Task Force on Clinical Applications of Bio-Markers [10, 11].

According to these criteria, most myocardial injuries detected during the perioperative period were acute. Considering that chronic myocardial injury is expected in a sizeable number of patients [23], it is likely that some chronic myocardial injuries might progress to acute conditions during the perioperative period. The variables associated with operative burden such as surgical risk, duration, and red blood cell transfusion were higher in the acute injury group. However, whether any of these variables are actually associated with induction of an acute injury from a chronic condition is beyond the scope of this study and needs a separate investigation that enrolls only patients who has chronic myocardial injury preoperatively. The preoperative variables showed that the patients with perioperative myocardial injury were older, more male, and had higher incidences of underlying diseases as previously reported [4–6, 9], but these demographic characteristics were not significantly different in the direct comparison between the acute and chronic injury groups.

Our results showed that postoperative mortalities of patients with acute and chronic myocardial injures were similarly high compared with patients without myocardial injury, and the mortality rates were not significantly different between the chronic injury and acute injury groups. This finding may have clinical implications for interpreting perioperative cTn measurements according to current recommended guidelines [7, 8, 14, 24]. Most evidence based on these guidelines highlights the importance of cTn changes and secondary prevention for patients with myocardial injury after noncardiac surgery [4, 18, 19, 25]. Meanwhile, our findings suggest that effective management of chronic myocardial injury is needed for surgical patients even when acute injury is not present. Whether additional perioperative management should be given to patients with chronic myocardial injury or whether the types of measurements that have been effective for acute myocardial injury patients after noncardiac surgery should be applied in chronic myocardial injury cases needs further investigation. Of note, traditional therapies for ischemic injury did not necessarily benefit myocardial injury patients with no evidence of infarction [26].

The overall incidence of perioperative myocardial injury in this study was higher than pervious reports [4–6, 9] This may be related to that we enrolled the patients with serial cTn measurements during the perioperative periods. Following the institutional protocol, our study patients were those who preoperatively had moderate to high risk and were postoperatively suspected of ischemic disease. Our institutional protocol may have applied a wide indication for cTn measurement compared with most centers in other countries. Although serial cTn measurements was recommended for high-risk patients by the Canadian society guideline [8], the clinical impact of applying the wide indication of cTn measurement remains unclear in this study. It is also unclear whether this wide indication for cTn measurement affected the result, so we conducted a statistical sensitivity analysis. In addition, difference between numbers of patients in the acute injury group and the chronic group was large. Therefore, for statistical adjustment in the comparison between those two groups, we chose IPW which is known to have benefit when there is a large difference in numbers [15].

The following limitations should be considered when interpreting the results of our study. First, as a single-center, observational study, our results might have been affected by selection bias or unmeasured confounding factors despite rigorous adjustments. Second, perioperative cTn was routinely measured for moderate- to high-risk patients, but was only selectively measured for mild-risk patients at the attending clinician’s discretion. Given that we enrolled patients with higher cardiovascular risk, our results may have been exaggerated, and during the study period of eight years, the institutional patient management including postoperative medication might have also changed. Also, in some of study patients, the baseline cTn value was measured after the surgery, and an elevation may not be directly related the surgical procedure. Third, the incidence of chronic injury was extremely low in the entire population, and the difference in prevalence between the two injury conditions was still broad after eliminating the normal group. The calculated study power based on the number of the chronic group was relatively low. Lastly, a detailed cardiac evaluation, including results for left ventricular ejection fraction or coronary artery angiograms, was not routinely performed. Despite these limitations, this is the first study to compare acute and chronic myocardial injury in surgical patients, demonstrating the similar impact on postoperative mortality between the two types of conditions.

## Conclusion

Chronic myocardial injury during the perioperative period may show similar clinical impacts on postoperative mortality as acute injury. Further studies on pathogenesis and treatment among these patients are needed.

## Supporting information

S1 Table Types of surgery. (DOCX)

S2 Table Baseline characteristics of the patients with perioperative myocardial injury. (DOCX)

S3 Table Sensitivity analysis of the effect of an unmeasured confounder on hazard ratio of the chronic myocardial injury group for 30-day mortality compared with the normal group. (DOCX)

## Abbreviations and acronyms

URL: upper reference limit
MINS: myocardial injury after noncardiac surgery
cTn: cardiac troponin
HR: hazard ratio
CI: confidence interval
IQR: interquartile range
