# Supplementary material for: Comparison of acute and chronic myocardial injury in noncardiac surgical patients
Source: PLoS One. 2020 Jul 2;15(7):e0234776. doi: 10.1371/journal.pone.0234776 (PMC7332041; doi:10.1371/journal.pone.0234776)
Supplement: S1 Table — (DOCX) [file pone.0234776.s001.docx]

S1 Table. Types of surgery.

|  | Normal | Acute myocardial injury | Chronic myocardial injury |
| --- | --- | --- | --- |
|  | (N=17671) | (N=5179) | (N=119) |
| Vascular | 1411 (8.0) | 559 (10.8) | 1 (0.8) |
| Orthopediatric | 1840 (10.4) | 766 (14.8) | 23 (19.3) |
| Neuro | 3518 (19.9) | 605 (11.7) | 13 (10.9) |
| Breast or Endo | 439 (2.5) | 106 (2.0) | 5 (4.2) |
| Plastic or Otolaryngeal or Eye | 537 (3.0) | 180 (3.5) | 10 (8.4) |
| Transplantation | 437 (2.5) | 487 (9.4) | 6 (5.0) |
| Gynecology or Urology | 1057 (6.0) | 341 (6.6) | 7 (5.9) |
| Gastrointestinal | 7078 (401) | 1302 (25.1) | 29 (24.4) |
| Noncardiac thoracic | 1301 (7.4) | 803 (15.5) | 23 (19.3) |
| Others | 53 (0.3) | 30 (0.6) | 2 (1.7) |
