# Supplementary material for: Comparison of acute and chronic myocardial injury in noncardiac surgical patients
Source: PLoS One. 2020 Jul 2;15(7):e0234776. doi: 10.1371/journal.pone.0234776 (PMC7332041; doi:10.1371/journal.pone.0234776)
Supplement: S2 Table — (DOCX) [file pone.0234776.s002.docx]

S2 Table. Baseline characteristics of the patients with perioperative myocardial injury

|  |  |  | Before IPW | | After IPW | |
| --- | --- | --- | --- | --- | --- | --- |
|  | Acute myocardial injury | Chronic myocardial injury | P-value | SMD | P-value | SMD |
|  | (N=5179) | (N=119) |  |  |  |  |
| Male | 3175 (61.3) | 74 (62.2) | 0.92 | 1.8 | 0.32 | 12.5 |
| Age | 65.7 (±13.8) | 64.1 (±14.0) | 0.21 | 11.7 | 0.56 | 5.8 |
| Diabetes | 2867 (55.4) | 70 (58.8) | 0.51 | 7 | 0.3 | 12.7 |
| Hypertension | 3484 (67.3) | 83 (69.7) | 0.64 | 5.3 | 0.66 | 5.1 |
| Current smoking | 483 (9.3) | 4 (3.4) | 0.04 | 24.7 | 0.21 | 16.6 |
| Current alcohol | 764 (14.8) | 15 (12.6) | 0.6 | 6.3 | 0.9 | 1.9 |
| Chronic kidney disease | 719 (13.9) | 28 (23.5) | 0.004 | 24.9 | 0.11 | 13.5 |
| History of ischemic heart disease | 1277 (24.7) | 25 (21.0) | 0.42 | 8.7 | 0.21 | 13.7 |
| History of heart failure | 201 (3.9) | 8 (6.7) | 0.18 | 12.7 | 0.08 | 11.3 |
| History of stroke | 508 (9.8) | 17 (14.3) | 0.14 | 13.8 | 0.69 | 3.8 |
| History of arrhythmia | 549 (10.6) | 12 (10.1) | 0.98 | 1.7 | 0.81 | 2.8 |
| History of heart valve disease | 107 (2.1) | 3 (2.5) | 0.99 | 3 | 0.86 | 1.5 |
| Active cancer | 2033 (39.3) | 45 (37.8) | 0.82 | 3 | 0.31 | 11.7 |
| Preoperative care |  |  |  |  |  |  |
| Intensive care unit | 610 (11.8) | 9 (7.6) | 0.2 | 14.3 | 0.88 | 2.3 |
| ECMO | 1 (0.0) | 0 | >0.99 | 2 | 0.88 | 1.9 |
| Continuous renal replacement therapy | 61 (1.2) | 0 | 0.45 | 15.4 | 0.23 | 15.1 |
| Ventilator | 140 (2.7) | 2 (1.7) | 0.69 | 7 | 0.75 | 5.2 |
| Operative variables |  |  |  |  |  |  |
| ESC/ESA surgical high risk | 1381 (26.7) | 11 (9.2) | <0.001 | 46.6 | 0.68 | 6.5 |
| Emergency operation | 1490 (28.8) | 36 (30.3) | 0.8 | 3.2 | 0.43 | 10.2 |
| General anesthesia | 4514 (87.2) | 95 (79.8) | 0.03 | 19.8 | 0.82 | 2.1 |
| Operation duration, hours | 3.53 (±2.79) | 2.52 (±2.11) | <0.001 | 40.9 | 0.99 | 0.2 |
| Continuous infusion of inotropics | 2217 (42.8) | 37 (31.1) | 0.01 | 24.5 | 0.23 | 15.1 |
| RBC transfusion | 826 (15.9) | 9 (7.6) | 0.02 | 26.3 | 0.75 | 5.2 |

IPW, inverse probability weighting; SMD, standardized mean difference; ECMO, extracorporeal membranous oxygenation; RAAS, renin-angiotensin-aldosterone system; ESC, European Society of cardiology; ESA, European Society of Anaesthesiology; RBC, red blood cell
