# Supplementary material for: Comparison of acute and chronic myocardial injury in noncardiac surgical patients
Source: PLoS One. 2020 Jul 2;15(7):e0234776. doi: 10.1371/journal.pone.0234776 (PMC7332041; doi:10.1371/journal.pone.0234776)
Supplement: S3 Table — (DOCX) [file pone.0234776.s003.docx]

S3 Table. Sensitivity analysis of the effect of an unmeasured confounder on hazard ratio of the chronic myocardial injury group for 30-day mortality compared with the normal group.

|  |  | OR*_ZY_*_\|_*_X_* | | | | | |
| --- | --- | --- | --- | --- | --- | --- | --- |
|  |  | 1.5 | 2 | 2.5 | 3 | 3.5 | 4 |
| OR_zx_ | 0.3 | 2.43 (1.54-3.84) | 2.62 (1.66-4.14) | 2.71 (1.72-4.28) | 3.04 (1.92-4.80) | 3.26 (2.05-5.16) | 3.33 (2.10-5.27) |
|  | 0.4 | 2.37 (1.50-3.74) | 2.59 (1.64-4.10) | 2.74 (1.73-4.32) | 2.86 (1.81-4.52) | 3.05 (1.93-4.83) | 3.15 (2.00-4.97) |
|  | 0.5 | 2.38 (1.51-3.76) | 2.52 (1.60-3.98) | 2.57 (1.63-4.06) | 2.86 (1.81-4.51) | 2.54 (1.61-4.01) | 3.05 (1.93-4.82) |
|  | 0.6 | 2.34 (1.48-3.70) | 2.32 (1.47-3.66) | 2.58 (1.64-4.08) | 2.55 (1.61-4.01) | 2.66 (1.69-4.19) | 3.16 (2.00-4.99) |
|  | 0.7 | 2.20 (1.40-3.48) | 2.25 (1.42-3.55) | 2.41 (1.53-3.80) | 2.71 (1.71-4.28) | 2.84 (1.80-4.48) | 2.75 (1.74-4.33) |

Prevalence of unmeasured confounder = 40%

Numbers represent HRs (including 95% CIs).

OR, odds ratio; HR, hazard ratio; x,: dichotomous exposure measure; y, dichotomous outcome measure; z, potential dichotomous confounder.

OR_ZX_ indicates the association (OR) between the unmeasured confounder and chronic myocardial injury.

OR_ZY|X_ indicates the association (OR) between the unmeasured confounder and mortality conditional on exposure status.
